# Health insurance coverage and health service utilisation in Uganda: an analysis of the 2016 Uganda Demographic and Health Survey

**DOI:** 10.1186/s12913-026-14900-9

**Published:** 2026-06-13

**Authors:** Susan Chinelo Ifeagwu, Maria Sarah Nabaggala, Carol Brayne, Rosalind Parkes-Ratanshi

**Affiliations:** 1 Department of Public Health and Primary Care, Cambridge Public Health, University of Cambridge, Cambridge, UK; 2 Infectious Diseases Institute, College of Health Sciences, Makerere University, Kampala, Uganda; 3 Department of Psychiatry, Cambridge Public Health, University of Cambridge, Cambridge, UK; 4 Infectious Diseases Institute, Makerere University College of Health Sciences, Kampala, Uganda

**Keywords:** Universal health coverage, Uganda, Health insurance, 2016 Uganda Demographic and Health Survey, 2016 UDHS, UHC, Quantitative

## Abstract

**Background:**

Health insurance is key in pooling resources for increased access to health services. There is currently no national health insurance scheme (NHIS) in Uganda and determining the profile of individuals with alternative health insurance can support future planning. The aim of this analysis was to explore the association between health insurance coverage and sociodemographic factors, health service utilisation and healthcare seeking behaviour in the 2016 Uganda Demographic and Health Survey (2016 UDHS).

**Methods:**

Descriptive statistics and bivariate Pearson’s chi-square tests of association were conducted for 18,506 women and 5,336 men sampled in the 2016 UDHS. Significant variables at *p* < 0.05 informed the multivariable mixed effects Poisson regression analysis; this examined associations between health insurance coverage, sociodemographic factors, health service utilisation, and knowledge of insurance. Analyses were carried out using R Programming.

**Results:**

Among the low proportion (1.4%) of individuals with health insurance, coverage widened with increasing wealth and education levels. Results of the regression analysis suggested a positive association between health service utilisation and being married, and increasing age, wealth, and knowledge of health insurance.

**Conclusions:**

Findings demonstrated a very low health insurance coverage among the 2016 UDHS. Adjusting for wealth and age, higher education levels were positively associated with coverage, indicating the importance of education in understanding health risk. Beyond an inclusive approach for vulnerable population groups, strategies for the NHIS need to incorporate sensitisation to stakeholders on the need for a personal health safety net. Further work on exploring how to ensure equity and financial risk protection in the NHIS implementation may be key to informal sector inclusion.

**Supplementary Information:**

The online version contains supplementary material available at 10.1186/s12913-026-14900-9.

## Background

“Good Health and Well-being” is embedded in the United Nations Sustainable Development Goals (UN SDGs). A key strategy of this is universal health coverage (UHC), defined by the World Health Organization (WHO) as all individuals and communities having access to any health services they need, of sufficient quality to be effective, in the absence of financial hardship [1, 2]. As a key component of UHC, health insurance schemes can be publicly or privately funded. In Uganda, a low-income country (LIC) in East Africa [3], health insurance is predominantly provided through private insurance schemes or employers. However, the number of individuals covered by health insurance remains low in the population. Many individuals in the population are still subject to high out-of-pocket (OOP) payments for healthcare services. This leads to an overall low UHC service coverage index of 50% [4–6]. 

The national health insurance scheme (NHIS) Bill was approved by the Parliament of Uganda in 2021 [7] and comprises social, community-based, and private health insurance; however, there are challenges in operationalisation. To support the rollout of the NHIS, obtaining information on past efforts and establishing the full profile of people that are already enrolled in health insurance schemes in the population are valuable. In addition, investigating profiles of individuals with health insurance coverage and how these relate to healthcare utilisation and seeking healthcare among the population could be informative. These insights could provide additional information to help introduce a future NHIS to the population in a sustainable way, considering potential challenges and next steps for implementation.

There have been various maternal healthcare studies based on individual Demographic and Health Survey (DHS) datasets in Africa, and these suggest variation in the effect of NHIS in improving access to health services and as a pro-poor strategy [8–14]. Kazungu and Barasa (2017) argued that in Kenya a tax funded mechanism should be adopted to provide health insurance to its population using a pre-payment mechanism [15]. An assessment of the level of health insurance coverage in 36 countries of Sub-Saharan Africa (SSA) using DHS datasets [16] showed that coverage levels were generally low at 7.9% and schemes were considered pro-rich. Four countries, Burundi, Gabon, Ghana, and Rwanda, with coverage levels above 20% [16], shared a common feature of being funding by tax revenues. Barasa et al. conclude that SSA countries would need to replace any existing voluntary schemes with tax funded mechanisms to tackle current inequalities in health insurance coverage [16]. 

In 2008, a systematic review conducted by Kiwanuka and colleagues identified a number of barriers to accessing and utilising healthcare in Uganda, arising both from service providers and consumers, namely distance to facilities, availability of drugs, perceived quality of care and lack of skilled staff, attitudes of health care workers (HCWs), costs of care, late referrals, and a general lack of knowledge [17]. Evidence suggests that if services are poor, convincing the population to commit to health insurance schemes is a challenge [18, 19]. Allcock and colleagues (2019) investigated the relationship between health insurance and service utilisation of the Namibian DHS 2011 [20]. Their findings revealed that of all sociodemographic factors associated with health insurance, education was considered the most significant positive association, suggesting the important role of this factor in health insurance coverage.

The 2016 Uganda Demographic and Health Survey (2016 UDHS) from the Demographic and Health Survey (DHS) Program, is a cross-sectional dataset and population-based survey of 19,588 households [21]. The majority of the population in Uganda is young, as more than 50% of the total population are children under the age of 15 [21]. Within the literature, evidence based on the UDHS datasets centres on maternal health insurance coverage and healthcare service utilisation in Uganda [22–24]. These studies looked at ante-natal, postnatal and delivery care, and maternal health services focusing on women of reproductive ages [22–24]. However, none of these studies examined the combined effects of health insurance, sociodemographic determinants, and healthcare service utilisation of the entire 2016 UDHS sample population. To fill this knowledge gap, this analysis aims to investigate the relationship between health insurance coverage, sociodemographic factors, and health service utilisation.

## Methods

### UDHS methodology

The data source for this analysis was the 2016 UDHS dataset. Data collection for the 2016 UDHS was conducted between 20 June and 16 December 2016, by the Uganda Bureau of Statistics (UBOS) and the Ugandan Ministry of Health (MoH); detailed methods are provided in the 2016 UDHS Report [25]. The national sampling frame was applied and specified by UBOS, which was the same as that of the 2014 Uganda Population and Housing Census (UPHC) [25]. 

For the 2016 UDHS, the 15 geographic regions were divided into 112 administrative districts and further sub-divided into enumeration areas (EAs) stratified by urban and rural areas, which were based on the UPHC census frame. EAs pertained to a village in rural areas and city block in urban areas and covered an average of 130 households [25]. In the first stage, 162 urban and 534 rural EAs or clusters were chosen. In the second stage, 30 households per cluster were selected independently with probability proportional to size [25]. Of these, 20,880 households were randomly selected and eligible for interviews. The final number of occupied households was 19,938 of which 19,588 were interviewed, yielding a response rate of 98%. In total, 173 fieldworkers, comprising 108 women and 65 men, were recruited, and trained by UBOS to conduct the questionnaires. Data collection was carried out by 21 teams.

### Eligibility criteria

Selected household members or visitors, aged 15–49 years for women, and 15–54 years for men, at the time of data collection, were included. Exclusion criteria comprised non-household members, and women aged < 15 or > 49 years, or men aged < 15 or > 54 years. In total, 18,506 women and 5,336 men (23,842 individuals) were included in the dataset.

### Extraction of data for this analysis

The 2016 UDHS is comprised of seven different recode files, which includes questionnaires for households, household members (persons), women, men, couples, births, and children. All members and visitors found at the selected households were included in the Household Questionnaire. The data for women and men were provided in the individual, and similarly labelled, Women’s and Men’s Questionnaires recode files.

Permission for access to the database was requested by the lead author on the Measure DHS Program website, and this was granted in 2020. For the purpose of this analysis, all recode files were downloaded and the files named “Household’s Questionnaire”, “Women’s Questionnaire” and “Men’s Questionnaire” were selected.

In both the Men’s and Women’s Questionnaires, respondents were asked whether they had health insurance coverage. Individuals were also asked which type of health insurance they were covered by. Health service uptake related questions were asked in the household survey. Two outcomes on healthcare service utilisation provided within the survey were used to examine the relationship between utilisation and health insurance coverage: “whether individuals had ever sought help” and “visited a health facility in the last 12 months”. Outcomes for whether an individual had “ever sought help” and “visited a health facility in the last 12 months” were coded into binary ‘yes’ or ‘no’ responses.

### Secondary data extraction and transformation

Data cleaning, manipulation and transformation were conducted using the Household, Women’s, and Men’s recode files. In total, 18,506 women and 5,336 men, comprising 23,842 individuals, were included in the analyses (Table 1). The three recode files (household, individual women, individual men) were merged into one file for further analyses through the line numbers. The sample weighting, which was derived by the DHS Program directly as a variable from each recode file, was applied for all frequency and proportion tabulations when representative statistics were computed. Both unweighted and weighted analyses were conducted to compare computed outcomes and ensure that the unweighted values were similar to weighted values and thus representative of the population. Additional analyses investigating relationships among variables were solely based on unweighted values, as the use of sample weights was deemed inapplicable for such analyses [25, 26]. 

The age of individuals was recoded into five-year group categorisations (15–19, 20–24, 25–29, 30–34, 35–39, 40–44, and 45–49 years) for women, and the same but an addition of 50–54 years for men (as no women respondents were in the 50–54 years range). For further detail into the type of health facility a household visited for healthcare, frequencies for the private and public sector were looked into, providing information into the type of health service utilised by the household. The private sector included private hospital/clinics, private doctors, private mobile clinics, pharmacies or drug shops and other private medical sector facilities. Public sector facilities included the government hospital, government health centres, family planning clinics, mobile clinics, and other public sector facilities. Outcomes of the background characteristics were displayed as frequencies and percentages. In certain cases, *p*-values were presented for the categorical data.

For the distribution of health insurance coverage among men and women, data were explored by sociodemographic factor. This was then combined to enable the assessment of all individuals covered by health insurance in the 2016 UDHS sample of respondents. To assess the variations in health insurance, the different types of health insurance, namely, employer-based insurance, mutual health organization or community-based insurance, privately purchased commercial insurance, social security and “other” were explored.

The categories for marital status included: never in union, married, living with partner, widowed, divorced, no longer living together/separated. For further analyses, this variable was recoded to a binary form: married and unmarried. The wealth index is a measure of a household’s cumulative living standard, calculated by data on ownership of assets, construction materials and sanitation facility access [27]. Developed using principal components analysis, the index is based on five wealth quintiles: lowest (1st), second (2nd), middle (3rd), fourth (4th), highest (5th).

As a proxy for health service utilisation, three variables were examined: individuals who had visited a health facility in the last 12 months, received medical help for self and ever sought help. In addition, the variable ‘considering joining a health insurance scheme’ was investigated as a potential proxy for willingness. The distribution of healthcare utilisation by sociodemographic characteristics was assessed, namely age, sex, health insurance, knowledge of health insurance, willingness to join a scheme, residence, region, education, literacy, employment, marital status, and wealth index. Frequency tabulations for the proxies for health service utilisation were presented.

Potential confounding sociodemographic variables were identified based on the literature and included age, education, literacy, marital status, sex, and wealth index or the cumulative wealth of a household [10, 20, 28, 29]. 

### Secondary data analysis

Analyses were conducted using R Programming version 3.6.1 and RStudio version 1.2.1335.

A bivariate Pearson’s chi-square test of association was carried out on selected sociodemographic variables (sex, age, residence, region, education, literacy, employment, being married, and wealth index) to examine the difference between the distribution of individuals who had health insurance against those who were not covered by health insurance. Significant variables at the *p*-value < 0.05 were then selected for further investigation in the Poisson regression analyses.

Poisson regression models were used because the outcome variable, health service utilisation represented count data, the number of occurrences of an event within a fixed time, which followed a Poisson distribution. The regression analyses included adjustments for covariates (age, sex, education, literacy, being married, wealth, employment, health insurance status and knowledge of health insurance) to avoid confounding bias. Coefficients were reported as risk ratios (RR) to enhance the interpretability of the results and ensure consistency with the model assumptions. Risk ratios were considered significant at a significance level of *p* < 0.05. Output of the Poisson regression analyses provided risk ratios with 95% confidence intervals.

Dependent variables in the analysis included individual health insurance coverage, measured by those who indicated “yes” to having health insurance. Independent variables included selected sociodemographic and economic variables (age, education, employment, knowledge of health insurance, literacy, marital status, region, residence type, sex, and wealth of respondents). Univariable Poisson regression analysis was conducted to examine the associations between health insurance and the selected sociodemographic and economic variables individually (Model 1). The multivariable Poisson regression analysis (Model 2) adjusted for all potentially confounding sociodemographic variables in relation to health insurance.

## Results

### Population characteristics

The main selected sociodemographic characteristics of the 2016 UDHS, stratified by sex, are displayed in Table 1. Of the total number of 23,842 individuals, over three quarters (77.6%) were women, with the mean and median ages for women and men both under 30 years (a mean of 27.9, 29.2 respectively, and a median of 26, 28 respectively). Nearly a quarter of both women and men were in the age group 15–19 (23%, 24% respectively). Most women and men lived in rural areas (73.3%, 75.1% respectively) and the highest proportion resided in South Buganda (Central). The lowest proportion of individuals resided in north-eastern Uganda, in Karamoja.

More individuals completing the questionnaire had only primary education (57.4% and 55.1%, respectively), whereas 7.9% of women and 12.5% of men had higher or tertiary education. Around 2,000 individuals had no education. More than half of all women and men had literacy (55.9% and 63.5% respectively), which was measured by those that were able to read whole sentences. Low literacy levels were also evident, with almost a third of women (31.4%) and one fifth of men (20.1%) not being able to read at all. Employment levels were relatively high, with most women and men employed at the time of the survey (73.1%, 92.3%, respectively). Less than a third of women were married, while slightly more than a third of men were married (30.3%, 36.1% respectively). While 25.8% of women and 39.0% of men were never in a union, an even lesser proportion of women and men were either divorced, no longer living together or separated, or widowed (13.5%, 5.6% respectively). Generally, the proportion or number of women and men respondents increased with increasing wealth quintiles.

**Table 1:** Selected sociodemographic characteristics of 2016 UDHS, stratified by sex (weighted values)

Background sociodemographic characteristics	Number of women and men combined (percentage, in %)	Number of women (percentage, in %)	Number of men (percentage, in %)
Age (in years)			
15–19	5,552 (23.3)	4,264 (23.0)	1,288 (24.1)
20–24	4,770 (20.0)	3,821 (20.6)	949 (17.8)
25–29	3,792 (15.9)	3,051 (16.5)	741 (13.9)
30–34	3,278 (13.7)	2,543 (13.7)	735 (13.8)
35–39	2,502 (10.5)	2,011 (10.9)	491 (9.2)
40–44	2,119 (8.9)	1,608 (8.7)	511 (9.6)
45–49	1,527 (6.4)	1,207 (6.5)	320 (6.0)
50–54	299 (1.3)	-	299 (5.6)
Residence			
Rural	14,893 (62.5)	13,563 (73.3)	4,006 (75.1)
Urban	8,949 (37.5)	4,943 (26.7)	1,330 (24.9)
Education			
Primary	13,570 (56.9)	10,630 (57.4)	2,940 (55.1)
Secondary	6,136 (25.7)	4,639 (25.1)	1,497 (28.1)
Higher/Tertiary	2,123 (8.9)	1,456 (7.9)	667 (12.5)
No education	2,004 (8.4)	1,781 (9.6)	223 (4.2)
Literacy			
Able to read only parts of sentence	3,039 (12.7)	2,215 (12.0)	824 (15.4)
Able to read whole sentence	13,743 (57.6)	10,353 (55.9)	3,390 (63.5)
No card with required language	135 (0.6)	98 (0.5)	37 (0.7)
Blind/visually impaired	38 (0.2)	29 (0.2)	9 (0.2)
Cannot read at all	6,886 (28.9)	5,811 (31.4)	1,075 (20.1)
Employment			
Currently employed	18,444 (77.4)	13,520 (73.1)	4,924 (92.3)
Not currently employed	5,398 (22.6)	4,986 (26.9)	412 (7.7)
Employed but absent	652 (2.7)	496 (2.7)	156 (2.9)
Marital status			
Never in union	6,866 (28.8)	4,783 (25.8)	2,083 (39.0)
Married	7,539 (31.6)	5,614 (30.3)	1,925 (36.1)
Living with partner	6,638 (27.8)	5,609 (30.3)	1,029 (19.3)
Widowed	542 (2.3)	522 (2.8)	20 (0.4)
Divorced	173 (0.7)	139 (0.8)	34 (0.6)
No longer living together/separated	2,084 (8.7)	1,839 (9.9)	245 (4.6)
Wealth index quintile			
Lowest	4,146 (17.4)	3,247 (17.5)	899 (16.8)
Second	4,344 (18.2)	3,397 (18.4)	947 (17.7)
Middle	4,507 (18.9)	3,459 (18.7)	1,048 (19.6)
Fourth	4,859 (20.4)	3,683 (19.9)	1,176 (22.0)
Highest	5,986 (25.1)	4,720 (25.5)	1,266 (23.7)
Total	23,842 (100.0)	18,506 (100.0)	5,336 (100.0)

Most respondents were aged 15 to 19 years (5,552, 23%). There were lower numbers of respondents as age increased. Individuals who were more affluent took part with most women (25.5%) and men (23.7%) being in the fifth and highest wealth quintile. However, the distribution of wealth indices is similar across the first to fourth quintiles, ranging from 17.5% to 19.9% for women and from 16.8% to 22.0% for men.

### Health insurance coverage

Overall, the percentage of individuals with health insurance in 2016 is approximately 6% for men and 5.6% for women, collectively. Table S1 (Appendix 1) displays the health insurance coverage distribution of the 2016 UDHS, by sociodemographic characteristics.

#### Profile of individuals covered by health insurance

**Fig. 1:**
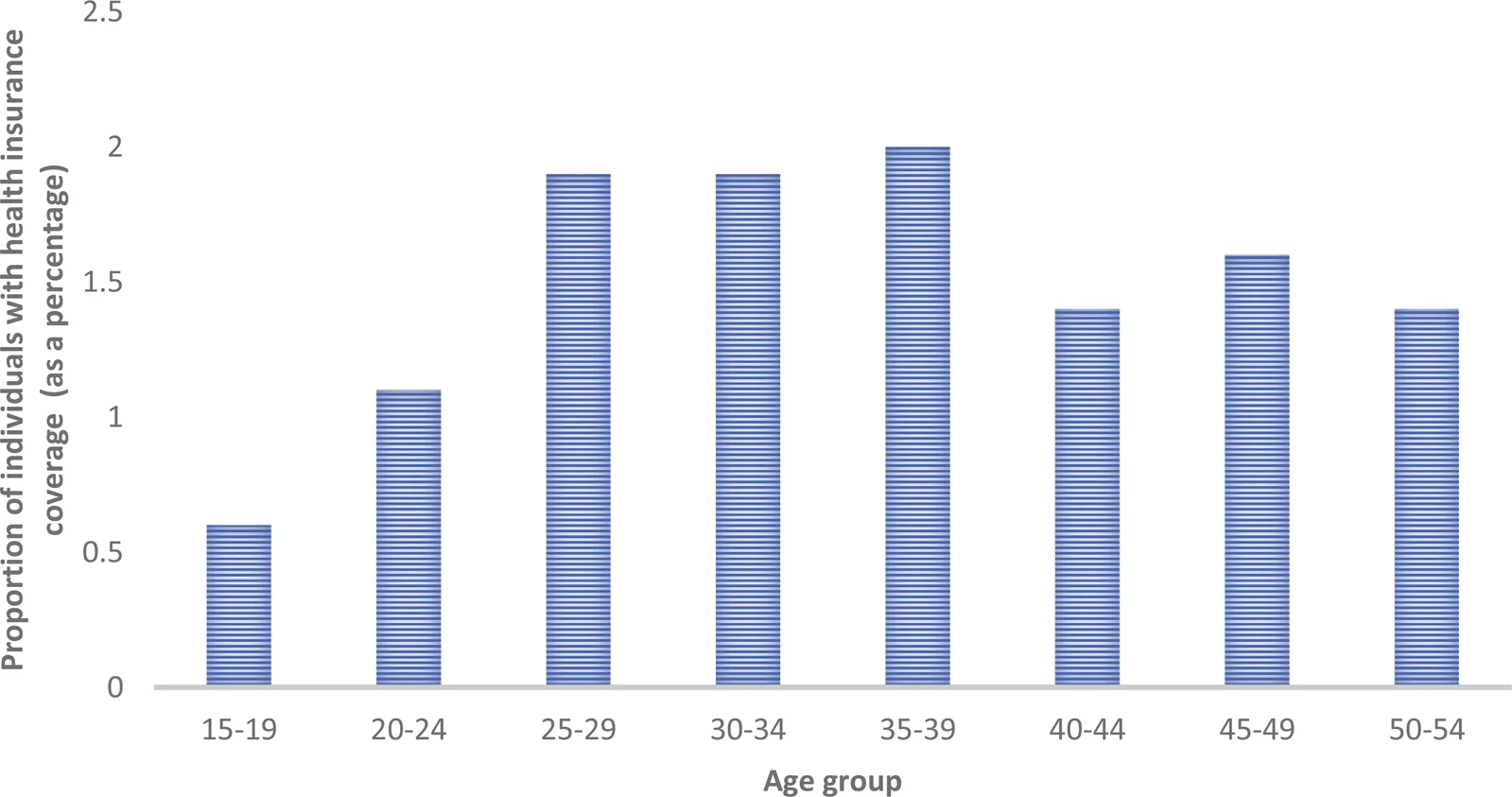
Proportion of health insurance coverage of the 2016 UDHS by age group

In general, health insurance coverage was low in the 2016 UDHS, with only 1.4% of individuals covered by health insurance (Fig. 1) with men (1.8%) higher than women (1.3%). As age increased, up to 39 years, reporting of health insurance increased (2.0%, *p* < 0.001), and thereafter declined with age. Health insurance coverage was higher in individuals living urban areas, 3.1%, as compared to rural areas, 0.9% (*p* < 0.001). The highest proportion of individuals resided in Kampala at 4.4% and Kigezi at 4.2% (*p* < 0.001).

Health insurance coverage increased with higher levels of education. The highest coverage was found in individuals with tertiary education at 7.4% compared to primary education at 0.6% (*p* < 0.001). Individuals with the highest literacy levels had greater health insurance coverage. A greater proportion of people covered by health insurance was found in individuals that were employed at the time of the survey compared to those who were not employed. Individuals who were married had higher levels of health insurance coverage (2.0%) compared to those who were not married (*p* < 0.001). A positive relationship was found between health insurance coverage and the wealth index quintiles of individuals. An increasing proportion of individuals had health insurance with increasing wealth, from 0.2% in the lowest wealth quintile to 3.8% in the highest wealth quintile, as would be expected given the cost of coverage (*p* < 0.001).

**Fig. 2:**
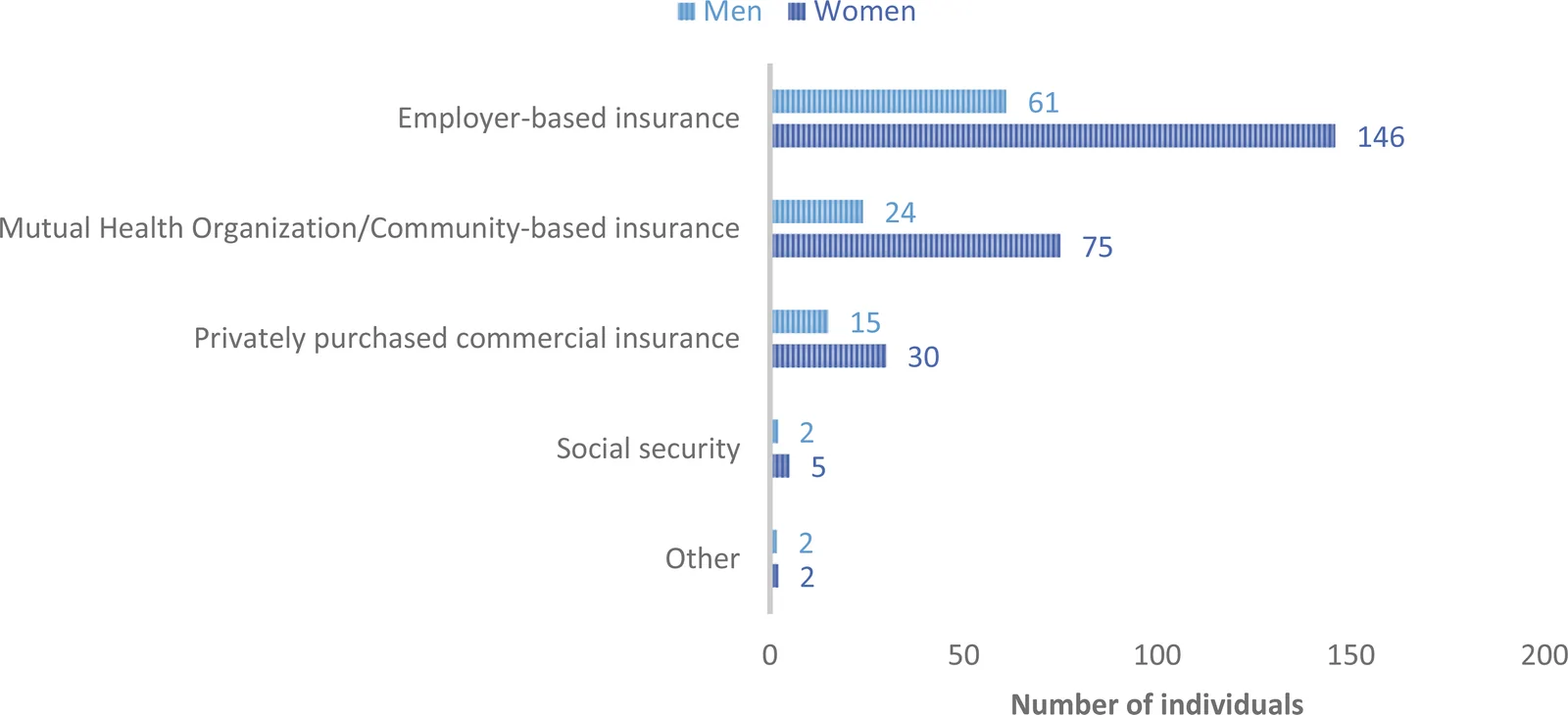
Specific type of health insurance among the 2016 UDHS

Of the subset of individuals with health insurance coverage, a majority of both men (*n* = 61 or 1.1%) and women (*n* = 146 or 0.8%) were covered through employer-based insurance (Fig. 2). The second largest type of health insurance mechanism was a mutual health organization or community-based insurance. Finally, the third most common type of health insurance was privately purchased commercial insurance. However, across all groupings, the total number of individuals covered by health insurance in the 2016 UDHS remained small.

### Health service utilisation

The two variables selected as health service utilisation proxies included individuals who had “ever sought help” and individuals who had visited a health facility in the last 12 months. Data was provided for both men and women, apart from the variable for having visited a health facility, which was missing in the *Men’s Questionnaire*. For the variable on whether individuals had ever sought help, a majority of data, more than 97% for both men and women, was not applicable. Thus, of the total responses only 0.3% of men and 0.5% of women reported that they had sought help. For the second health utilisation variable, more than two thirds, *n* = 12,572, thus 68.9%, of women reported to have visited a health facility in the last 12 months.

Most households, 72.6%, reported they sought health services from the public sector (14,237 out of 19,588). Of these, most of the households (61.8%) visited government health centres, followed by government hospitals (10.7%), and to a lesser extent family clinics, mobile clinics, and other public sector facilities. Of the 27.2% (*n* = 5,351) who visited private sector facilities for their healthcare needs, 24.8% used private hospitals or clinics, and to a lesser extent, private doctors, private mobile clinics, pharmacies or drug shops, and other private sector facilities.

#### Association between health service utilisation distribution, health insurance coverage and sociodemographic factors

Health service utilisation and the relationship to related sociodemographic factors is presented in Table 2. In total, *n* = 12,770 (68.4%) of individuals utilised healthcare services, the majority (12,752, 68.9%) of which were women. For the individuals who had health insurance coverage, 77.5% utilised health services, while 22.5% did not. Of the individuals without health insurance coverage, 68.3% utilised health services (*p =* 0.002). Overall, a higher proportion of individuals covered by health insurance utilised health services as opposed to individuals without health insurance coverage. Those aged 25–29 years reported the highest healthcare utilisation. Among the other sociodemographic factors, a higher proportion of individuals in Karamoja (91.8%, *p* < 0.001), who were married (79.0%, *p* < 0.001), had higher/tertiary education (72.6%, *p* < 0.001) and were in the lowest wealth quintile (74.8%, *p* < 0.001) utilised healthcare services.

**Table 2:** Distribution of healthcare utilisation by sociodemographic characteristics (*n* = 18,664, unweighted values)

Sociodemographic characteristics	Healthcare utilisation (individuals who have ever sought help or visited a health facility in last 12 months)		
	Yes (%)	No (%)	*p*
Health insurance coverage			
Yes	186 (77.5)	54 (22.5)	0.002
No	12,584 (68.3)	5,840 (31.7)	
Know about health insurance			
Yes	3,064 (73.2)	1,121 (26.8)	< 0.001
No	9,706 (67.0)	4,773 (33.0)	
Would consider joining a health insurance scheme			
Yes	2,477 (73.5)	894 (26.5)	0.08
No	402 (69.9)	173 (30.1)	
Age range (in years)			
15–19	2,205 (51.2)	2,104 (48.8)	< 0.001
20–24	2,798 (73.2)	1,023 (26.8)	
25–29	2,349 (77.2)	692 (22.8)	
30–34	2,001 (76.2)	626 (23.8)	
35–39	1,483 (72.9)	552 (27.1)	
40–44	1,116 (68.4)	515 (31.6)	
45–49	815 (68.6)	373 (31.4)	
50–54	3 (25.0)	9 (75.0)	
Residence			
Rural	9,868 (69.3)	4,380 (30.7)	< 0.001
Urban	2,902 (65.7)	1,514 (34.3)	
Education			
Primary	7,481 (68.1)	3,508 (31.9)	< 0.001
Secondary	2,848 (66.8)	1,414 (33.2)	
Higher/Tertiary	973 (72.6)	367 (27.4)	
No education	1,468 (70.8)	605 (29.2)	
Literacy			
Able to read only parts of sentence	1,489 (65.8)	773 (34.2)	< 0.001
Able to read whole sentence	6,767 (68.3)	3,141 (31.7)	
No card with required language	55 (55.0)	45 (45.0)	
Blind/visually impaired	22 (64.7)	12 (35.3)	
Cannot read at all	4,437 (69.8)	1,923 (30.2)	
Employment			
Currently employed	9,974 (72.2)	3,846 (27.8)	< 0.001
Not currently employed	2,796 (57.7)	2,048 (42.3)	
Current marital status			
Never in union	2,312 (48.3)	2,479 (51.7)	< 0.001
Married	4,638 (79.0)	1,233 (21.0)	
Living with partner	4,124 (73.6)	1,477 (26.4)	
Widowed	369 (70.4)	155 (29.6)	
Divorced	101 (72.7)	38 (27.3)	
No longer living together/separated	1,226 (70.5)	512 (29.5)	
Wealth index quintile			
Lowest	2,929 (74.8)	986 (25.2)	< 0.001
Second	2,537 (69.2)	1,130 (30.8)	
Middle	2,367 (67.1)	1,158 (32.9)	
Fourth	2,329 (66.9)	1,152 (33.1)	
Highest	2,608 (64.0)	1,468 (36.0)	
Total	12,770 (68.4)	5,894 (31.6)	

Note: *p*-value indicates a chi-square test

### Poisson regression analysis

A Poisson regression analysis was conducted to further examine the association between health service utilisation and health insurance in relation to the selected sociodemographic and related factors. Table 3 presents the risk ratio (RR) and 95% confidence intervals. Model 1 displays the univariable associations between these factors, whereas Model 2 presents the multivariable Poisson regression adjusted for all potentially confounding sociodemographic variables. Of the entire 2016 UDHS, 12,770 (68.4%) sought care or accessed health services, indicating health service utilisation.

**Table 3:** Association between selected factors and health service utilisation among men and women (*n* = 12,770)

Selected sociodemographic and related factors	Model 1 - Univariable associations		Model 2 - Multivariable Poisson regression*	
	RR (95% CI)	*p*-value	RR (95% CI)	*p*-value
Age group				
15–19	1.00 (reference)		1.00 (reference)	
20–24	1.43 (1.35–1.51)	< 0.001	1.14 (1.07–1.22)	< 0.001
25–29	1.51 (1.42–1.60)	< 0.001	1.13 (1.06–1.22)	< 0.001
30–34	1.49 (1.40–1.58)	< 0.001	1.10 (1.02–1.18)	0.01
35–39	1.42 (1.33–1.52)	< 0.001	1.05 (0.97–1.14)	0.23
40–44	1.34 (1.24–1.44)	< 0.001	0.99 (0.91–1.07)	0.76
45–49	1.34 (1.24–1.45)	< 0.001	0.98 (0.90–1.08)	0.75
50–54	0.49 (0.12–1.27)	0.22	2.34 (0.54–7.11)	0.22
Sex				
Male	1.00 (reference)		1.00 (reference)	
Female	6.05 (3.94–9.98)	< 0.001	6.90 (4.33–12.00)	< 0.001
Health insurance				
No	1.00 (reference)		1.00 (reference)	
Yes	1.13 (0.98–1.31)	0.09	1.03 (0.88–1.19)	0.73
Education level				
No education	1.00 (reference)		1.00 (reference)	
Primary	0.96 (0.91–1.02)	0.17	1.01 (0.95–1.07)	0.81
Secondary	0.94 (0.89–1.01)	0.07	1.04 (0.96–1.13)	0.31
Higher	1.03 (0.95–1.11)	0.54	1.07 (0.97–1.19)	0.17
Literacy level				
Cannot read at all	1.00 (reference)		1.00 (reference)	
Able to read only parts of sentence	0.94 (0.89–1.00)	0.05	1.01 (0.95–1.08)	0.74
Able to read whole sentence	0.98 (0.94–1.02)	0.27	1.07 (1.02–1.12)	0.001
No card with required language	0.79 (0.60–1.02)	0.08	0.82 (0.62–1.06)	0.15
Blind/visually impaired	0.93 (0.59–1.37)	0.72	1.04 (0.66–1.54)	0.87
Marital status				
Never in union	1.00 (reference)		1.00 (reference)	
Married	1.64 (1.56–1.72)	< 0.001	1.54 (1.44–1.64)	< 0.001
Living with partner	1.53 (1.45–1.61)	< 0.001	1.43 (1.34–1.52)	< 0.001
Widowed	1.46 (1.31–1.63)	< 0.001	1.43 (1.27–1.62)	< 0.001
No longer living together/separated	1.46 (1.36–1.57)	< 0.001	1.37 (1.27–1.49)	< 0.001
Divorced	1.51 (1.23–1.83)	< 0.001	1.44 (1.17–1.76)	< 0.001
Wealth quintile				
Lowest	1.00 (reference)		1.00 (reference)	
Second	0.92 (0.88–0.98)	0.001	0.92 (0.87–0.97)	0.001
Middle	0.90 (0.85–0.95)	< 0.001	0.89 (0.85–0.95)	< 0.001
Fourth	0.89 (0.85–0.94)	< 0.001	0.87 (0.83–0.93)	< 0.001
Highest	0.86 (0.81–0.90)	< 0.001	0.83 (0.78–0.88)	< 0.001
Employment				
Unemployed	1.00 (reference)		1.00 (reference)	
Employed	1.25 (1.20–1.30)	< 0.001	1.11 (1.06–1.16)	< 0.001
Knowledge of health insurance				
No	1.00 (reference)		1.00 (reference)	
Yes	1.09 (1.05–1.14)	< 0.001	1.10 (1.05–1.15)	< 0.001

RR: Risk ratio from Poisson regression analysis; 95% CI: 95% Confidence intervals

Model 2: Adjusted for all confounding variables within the table

Individuals aged between 20 and 24 years were 14% more likely to report use of health services than other age groups (Model 2, RR: 1.14, 95% CI: 1.07–1.22; *p* < 0.001). Similarly, individuals aged between 25 and 29 years were 13% more likely to utilise health services (RR: 1.13, 95% CI: 1.06–1.22; *p* < 0.001). Women participants were more likely to report use of health services than men (Model 2, RR: 6.90, 95% CI: 4.33 -12.00; *p* < 0.001). Those with higher literacy levels were more likely to report use of health services, with individuals who were able to read whole sentences being 7% more likely to access services. Married individuals had the highest likelihood of reporting health service usage (Model 2, RR: 1.54, 95% CI: 1.44–1.64; *p* < 0.001). Being employed and having knowledge of health insurance were also significantly associated (Model 2, RR: 1.11, 95% CI: 1.06–1.16; *p* < 0.001 and RR: 1.10, 95% CI: 1.05–1.15; *p* < 0.001, respectively).

There was a negative relationship between the wealth index and reported health service utilisation. Individuals in the highest wealth quintile were 17% less likely to report use of health services (Model 2, RR: 0.83, 95% CI: 0.78–0.88; *p* < 0.001). Of all variables, the associations between health insurance, age groups of 35 to 54 years, and education level were not significant (*p* > 0.05). With increasing education, individuals were more likely to access health services. A likelihood ratio test of fit of Model 2 and a reduced model demonstrated that Model 2 showed a significantly better fit.

**Fig. 3:**
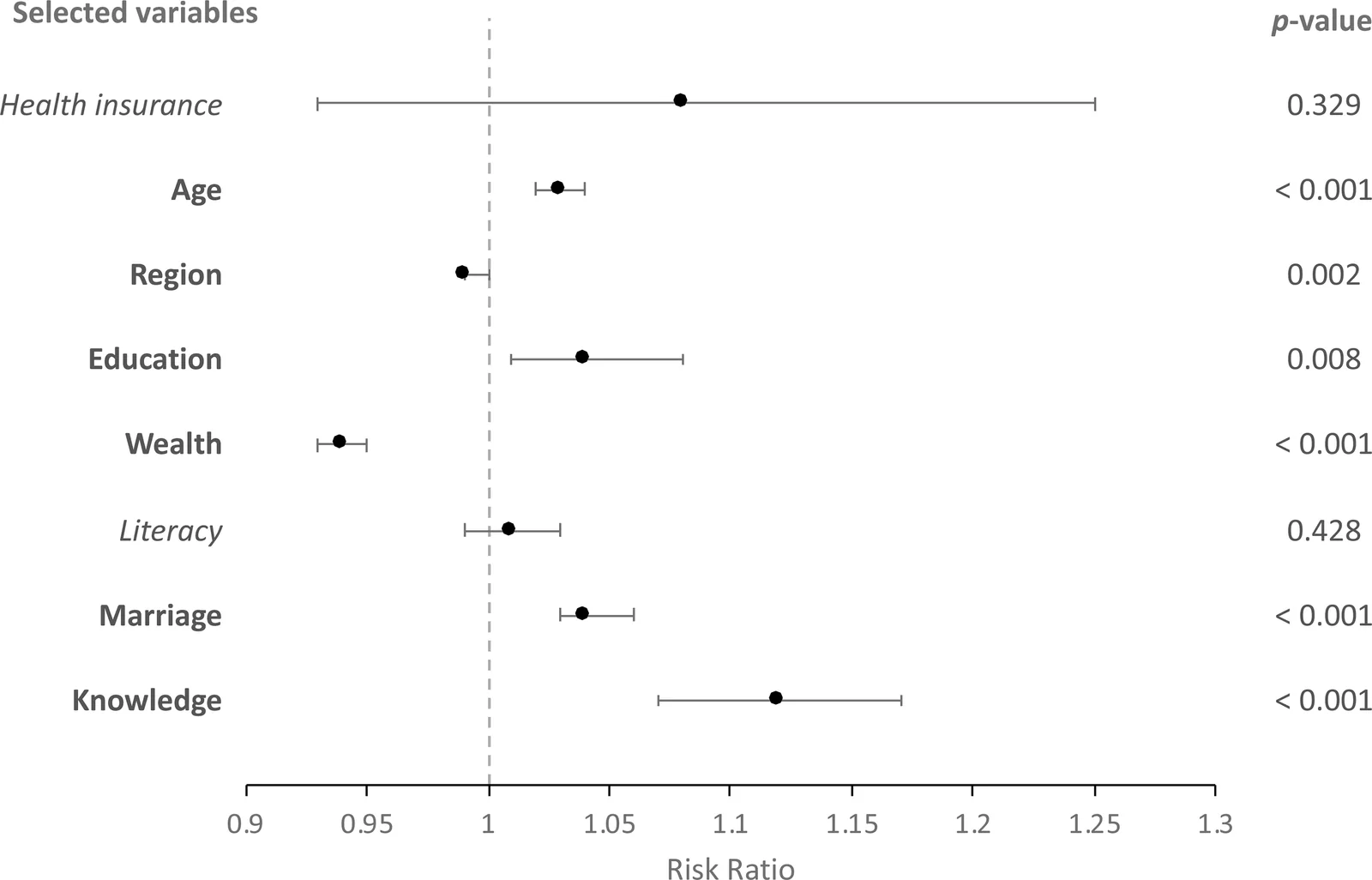
Forest plot of the association between health service utilisation, health insurance and sociodemographic factors

Results of the overall association between reported health service utilisation and sociodemographic factors showed that age (RR: 1.03, 95% CI: 1.02–1.04; *p* < 0.001), region (RR: 0.99, 95% CI: 0.99-1.00; *p* = 0.002), education (RR: 1.04, 95% CI: 1.01–1.08; *p* = 0.008), wealth (RR: 0.94, 95% CI: 0.93–0.95; *p* < 0.001), marriage (RR: 1.04, 95% CI: 1.03–1.06; *p* < 0.001), and knowledge (RR: 1.12, 95% CI: 1.07–1.17; *p* < 0.001) were significant (Fig. 3). Region and wealth of individuals were less likely to affect utilisation of health services. Age, education, marriage, and knowledge were positively associated with reported health service utilisation. Health insurance was not significantly associated with reported use of health services (RR: 1.08, 95% CI: 0.93–0.95; *p* = 0.329).

For individuals with health insurance coverage, significantly associated sociodemographic factors included age (RR: 1.17, 95% CI: 1.10–1.23; *p* < 0.001), education (RR: 2.29, 95% CI: 2.0-2.6; *p* < 0.001), region (RR: 1.04, 95% CI: 1.02–1.07; *p* < 0.001) and wealth (RR: 1.66, 95% CI: 1.48–1.86; *p* < 0.001). Individuals with higher education were 2.3 times more likely to have health insurance (Table S2 in Appendix 2).

## Discussion

This analysis revealed an overall very low health insurance coverage among the 2016 UDHS, where solely 1.4% of the total number of individuals were covered by insurance. Of those who were insured, coverage increased with rising wealth and education level. Most individuals were uninsured, prompting further negative implications for accessing health services. Findings of the regression analysis suggested an association between health insurance and health service utilisation. There is a trend towards the likelihood of accessing services in insured individuals being higher than in those who were not insured, although not reaching traditional significance. Marital and employment status and knowledge of health insurance were significantly associated with reported increased health service utilisation.

### Health insurance coverage and sociodemographic determinants

Consistent with earlier studies in Africa [20, 30], a marginally higher proportion of health insurance coverage was found in men compared with women (1.8% and 1.3%, respectively). This could be due to response bias whereby a smaller number of men participating in the survey were those who were more likely to have health insurance. A health insurance coverage study among women in East Africa between 2010 and 2018 (Weldesenbet et al. in 2021) reported an overall prevalence of 7.56%, with the lowest coverage found in Uganda (1.38%), concurring with this analysis [31]. Of the individuals who had health insurance, most were insured through employer-based schemes, a finding which is corroborated in the literature, where individuals working in the formal sector were covered through insurance schemes [15, 32]. A smaller proportion of individuals was covered by a mutual health organization or community-based health insurance (CBHI) and an even smaller proportion through privately purchased commercial insurance. The early mid-life group were those with the highest coverage by health insurance, but still a very small proportion 35–39 (2%) (Table S1, Appendix 1). Given the type of health insurance, most individuals who were insured lived in urban areas (3.1%), particularly in Kampala, as opposed to rural areas, thus implying greater affluence in those that were insured.

With increasing education levels, more individuals were covered by health insurance and those with higher or tertiary education were proportionally the most insured individuals (7.4%), which was a consistent finding among previous research conducted in Africa [15, 16, 30, 33]. Most of the individuals with health insurance coverage had high literacy levels compared to those with lower levels. As other regional studies have also demonstrated, individuals who were employed, married and within the highest wealth quintile were the most insured [31, 33, 34]. Therefore, these sociodemographic factors were closely related to whether or not a person was covered by health insurance, suggesting greater affordability among this group of individuals. In contrast, individuals who were unemployed, widowed, divorced, or not married, and in the lowest wealth quintile were the least covered by health insurance.

Despite low health insurance coverage, a greater number reported their awareness and willingness in joining such insurance schemes. A higher proportion of men were willing to join a health insurance scheme than women (28% versus 19%, respectively). Among both men and women, more individuals had knowledge of health insurance than were willing to participate in a scheme. However, there was a large amount of missing data on willingness for all individuals, thus this factor was not explored further.

A tiny proportion of men (0.09%) had accessed healthcare in the last 12 months. Most individuals of both genders with health insurance (*n* = 186, 77.5%) reported that they accessed such services. Notably the proportion of reported health service utilisation was higher among insured than non-insured individuals. However, contrary to previous studies [31, 35], a lack of health insurance was not a deterrent to accessing services in the respondents, as most individuals without coverage still reported high levels of health service utilisation (68.3%). Within the literature, the overall relationship between health insurance coverage and healthcare utilisation in the region is not definitive, in part due to issues with data availability and infrequent data reporting [36]. The highest proportion of those who reported use of health services lived in Karamoja, were married, had higher or tertiary education and were within the lowest wealth quintile.

The findings of the regression analysis revealed an association between reported health service utilisation and age, marital status, wealth, and knowledge of health insurance. Particularly, younger women aged between 20 and 34 years with high literacy levels, within high wealth quintiles, who had employment, were married, and had knowledge of health insurance were more likely to report use of health services. These results indicated a greater usage of health services among younger individuals potentially given more frequent health seeking behaviours related to increased antenatal or postnatal check-ups [37, 38]. In contrast to other findings, there was a negative relationship between the wealth index of individuals and reported health service utilisation, as those in the highest wealth quintile were less likely to report use of health services compared to individuals in the lowest quintile [16, 37]. This could have been due to the influence of other factors beyond wealth and poverty, such as employment status and overall higher levels of health due to better nutrition, living and working conditions [6, 39, 40]. Individuals with health insurance were 3% more likely to report use of healthcare services than those without coverage, however, this association was not significant.

Outcomes of this study could help support stakeholders in the planning and implementation phases of the NHIS roll out in future, by detailing the profile of individuals with health insurance coverage within this cohort and examining the association between individuals covered by health insurance and health service utilisation. Given that health insurance is an important element of UHC which helps to support financial risk protection of individuals, a greater understanding of insurance coverage and related sociodemographic factors could help support equity in schemes and add to knowledge for the implementation stage of a future NHIS in Uganda.

### Limitations

The country case study approach prompts issues of generalisability of findings to other LICs. However, the focus on Uganda sought to contribute to the general understanding of health insurance coverage in low-resource settings. Some of the main limitations of the 2016 UDHS sample of respondents surveyed, which impacted the findings, were a lack of relationship between the true population and survey population, and the distribution of individuals, as women comprised almost 80%. Since a greater proportion of women answered the survey than men, response, participation, and interpretation bias had to be considered as results were skewed towards women. Age bias was also apparent, given the absence of a higher age group for women and no people aged 60 years and above. Interpretations may not be fully representative of the wider population.

Although this is one of the most comprehensive global health datasets available, the surveys were tailored to maternal and child health indicators as opposed to a balance of adult health indicators, and consequently the breadth of data collected within the Men’s Questionnaire was more limited than the Women’s Questionnaire. Health service utilisation indicators were not equal across the different datasets, in part due to the survey design and initial focus of the DHS Program on generating maternal and child health data. Therefore, indicators related to reported health service utilisation, such as whether or not an individual visited a health facility in the last 12 months, were not included in the “Men’s Questionnaire”, compounding the issue of data skewedness. Associations between factors could have been impacted by additional confounding factors besides those explored in the analysis or beyond the scope of the dataset.

Finally, data was collected more than seven years ago, thus interpretations may not reflect the present context in Uganda. There is an ongoing updated survey but this is not yet published. The risk of limitations was reduced through conducting a likelihood ratio test of fit and by developing careful interpretations throughout. Therefore, it was decided to proceed with the study to provide analyses rather than none. Despite these limitations, some important findings concerning the relationship of health insurance coverage, sociodemographic factors, and health service utilisation were found.

## Conclusions

In conclusion, health insurance coverage among the 2016 UDHS was very low and fragmented, with differences in types of insurance schemes, populations and services covered and financial resources. Results of the Poisson regression suggested a positive association between health service utilisation and age, marital status, wealth, and knowledge of health insurance. Major inequalities were clearly present. Despite issues of data representativeness, the findings support the issue of a lack of health insurance coverage among the population of Uganda. This absence in coverage compounds the problem of individuals potentially not seeking needed healthcare due to high OOPs, and in some cases may lead to catastrophic health expenditure of households, further exacerbating health issues. While a public NHIS has yet to be implemented in the country, as opposed to other countries in the region, equity, affordability, and financial risk protection need to remain at its core. Inclusion of the informal sector in an equitable way, to avoid detrimental health inequities through unequal access to health insurance, will need to be ensured for the implementation of a sustainable NHIS.

## Supplementary Information

Below is the link to the electronic supplementary material.

Supplementary Material 1

## Data Availability

The data that support the findings of this study are available from the Demographic and Health Surveys (DHS) Program but restrictions apply to the availability of these data, which were used under license for the current study, and so are not publicly available. Data are however available from the authors upon reasonable request and with permission of the DHS Program, available at https://www.dhsprogram.com/.

## Abbreviations

95% CI: 95% Confidence Interval
CBHI: Community-Based Health Insurance
DHS: Demographic and Health Survey
EA: Enumeration Area
HCW: Health Care Worker
LIC: Low-income country
MoH: Ministry of Health
NHIS: National health insurance scheme
OOP: Out-of-pocket
RR: Risk Ratio
SDGs: Sustainable Development Goals
SSA: Sub-Saharan Africa
UBOS: Uganda Bureau of Statistics
UDHS: Uganda Demographic and Health Survey
UHC: Universal health coverage
UN: United Nations
UPHC: Uganda Population and Housing Census
WHO: World Health Organization
