# Supplementary material for: Health insurance coverage and health service utilisation in Uganda: an analysis of the 2016 Uganda Demographic and Health Survey
Source: BMC Health Serv Res. 2026 Jun 13;26:1167. doi: 10.1186/s12913-026-14900-9 (PMC13491677; doi:10.1186/s12913-026-14900-9)
Supplement: Supplementary file 1 [file 12913_2026_14900_MOESM1_ESM.docx]

# Supplementary Material

# Appendices

## Appendix 1.

Table S1. Health insurance coverage distribution of 2016 UDHS population, by sociodemographic characteristics (*n*=23,509, unweighted values)

| Background sociodemographic characteristics | Covered by health insurance (percentage in %) | | |
| --- | --- | --- | --- |
|  | Yes (%) | No (%) | *p* |
| Sex |  |  |  |
| Men | 94 (1.8) | 5,242 (98.2) | 0.01 |
| Women | 239 (1.3) | 18,267 (98.7) |  |
|  |  |  |  |
| Age (in years) |  |  |  |
| 15-19 | 31 (0.6) | 5,515 (99.4) | < 0.001 |
| 20-24 | 54 (1.1) | 4,672 (98.9) |  |
| 25-29 | 73 (1.9) | 3,681 (98.1) |  |
| 30-34 | 65 (1.9) | 3,272 (98.1) |  |
| 35-39 | 51 (2.0) | 2,475 (98.0) |  |
| 40-44 | 30 (1.4) | 2,083 (98.6) |  |
| 45-49 | 25 (1.6) | 1,522 (98.4) |  |
| 50-54 | 4 (1.4) | 289 (98.6) |  |
|  |  |  |  |
| Residence |  |  |  |
| Rural | 164 (0.9) | 18,149 (99.1) | < 0.001 |
| Urban | 169 (3.1) | 5,360 (96.9) |  |
|  |  |  |  |
| Region |  |  |  |
| Kampala | 72 (4.4) | 1,578 (95.6) | < 0.001 |
| South Buganda (Central) | 51 (2.5) | 2,012 (97.5) |  |
| North Buganda (Central) | 25 (1.3) | 1,845 (98.7) |  |
| Busoga | 11 (0.6) | 1,963 (99.4) |  |
| Bukedi | 11 (0.7) | 1,554 (99.3) |  |
| Bugisu | 7 (0.6) | 1,245 (99.4) |  |
| Teso | 5 (0.3) | 1,693 (99.7) |  |
| Karamoja | 7 (0.8) | 892 (99.2) |  |
| Lango | 7 (0.4) | 1,650 (99.6) |  |
| Acholi | 7 (0.5) | 1,455 (99.5) |  |
| West Nile | 7 (0.4) | 1,592 (99.6) |  |
| Bunyoro | 11 (0.7) | 1,547 (99.3) |  |
| Tooro | 24 (1.4) | 1,684 (98.6) |  |
| Ankole | 37 (2.2) | 1,644 (97.8) |  |
| Kigezi | 51 (4.2) | 1,155 (95.8) |  |
|  |  |  |  |
| Education |  |  |  |
| Primary | 79 (0.6) | 13,861 (99.4) | < 0.001 |
| Secondary | 93 (1.6) | 5,569 (98.4) |  |
| Higher/Tertiary | 143 (7.4) | 1,795 (92.6) |  |
| No education | 18 (0.8) | 2,284 (99.2) |  |
|  |  |  |  |
| Literacy |  |  |  |
| Able to read only parts of sentence | 15 (0.5) | 3,083 (99.5) | < 0.001 |
| Able to read whole sentence | 269 (2.0) | 12,853 (98.0) |  |
| No card with required language | 5 (3.8) | 125 (96.2) |  |
| Blind/visually impaired | 1 (2.3) | 43 (97.7) |  |
| Cannot read at all | 43 (0.6) | 7,405 (99.4) |  |
|  |  |  |  |
| Employment |  |  |  |
| Currently employed | 284 (1.5) | 18,291 (98.5) | 0.001 |
| Not currently employed | 49 (0.9) | 5,218 (99.1) |  |
|  |  |  |  |
| Current marital status |  |  |  |
| Never in union | 77 (1.1) | 6,690 (98.9) | < 0.001 |
| Married | 157 (2.0) | 7,708 (98.0) |  |
| Living with partner | 76 (1.2) | 6,450 (98.8) |  |
| Widowed | 4 (0.7) | 541 (99.3) |  |
| Divorced | 1 (0.6) | 172 (99.4) |  |
| No longer living together/separated | 18 (0.9) | 1948 (99.1) |  |
|  |  |  |  |
| Wealth index quintile |  |  |  |
| Lowest | 9 (0.2) | 4,932 (99.8) | < 0.001 |
| Second | 25 (0.5) | 4,662 (99.5) |  |
| Middle | 39 (0.9) | 4,495 (99.1) |  |
| Fourth | 64 (1.4) | 4,473 (98.6) |  |
| Highest | 196 (3.8) | 4,947 (96.2) |  |
|  |  |  |  |
| Total | 333 (1.4) | 23,509 (98.6) |  |
| *Note: p-value indicates the chi-square test* | |  |  |

## Appendix 2.

Table S2. Overall association between health insurance and sociodemographic factors

| Sociodemographic factors | RR (95% CI) | *p*-value |
| --- | --- | --- |
| Age | 1.17 (1.10 – 1.23) | < 0.001 |
| Region | 1.04 (1.02 – 1.07) | < 0.001 |
| Education | 2.29 (1.98 – 2.64) | < 0.001 |
| Wealth index | 1.66 (1.48 – 1.86) | < 0.001 |

RR: Risk ratio from poisson regression analysis; 95% CI: 95 % Confidence intervals.
